# Functional alterations of peripheral blood CD4+ T lymphocyte subsets in patients with Parkinson disease: A review

**DOI:** 10.1097/MD.0000000000048500

**Published:** 2026-05-01

**Authors:** Le Wang, Ruijun Su

**Affiliations:** a Department of Clinical Laboratory Diagnosis, Affiliated Hospital of Inner Mongolia Medical University, Hohhot, China.

**Keywords:** CD4+T cells, helper T cells, immune response, Parkinson disease, regulatory T cells

## Abstract

Parkinson disease (PD) is a neurological degenerative disorder common among the elderly. Neuroinflammation leads to neurodegeneration, which is regulated by the peripheral adaptive immunity. This review discusses the regulatory roles of neuroinflammation by CD4 + T cell subsets (T helper [Th] cell 1, Th2, Th17, Th9, and regulatory T cells). First, we emphasized the roles and mechanisms of CD4 + T cell subsets, as well as the cytokines they secrete, in PD. Second, we explore the associations between helper T cells, regulatory T cells, core cytokines (interleukin 2, 6, 8, tumor necrosis factor-α, interferon-γ), and chemokines (monocyte chemoattractant protein-1) and PD and clarify the role of peripheral inflammation in disease progression. Finally, analyzing the operation of CD4 + T cell subsets in the nervous system will facilitate the development of new therapeutic interventions.

## 1. Introduction

**Figure 1.**
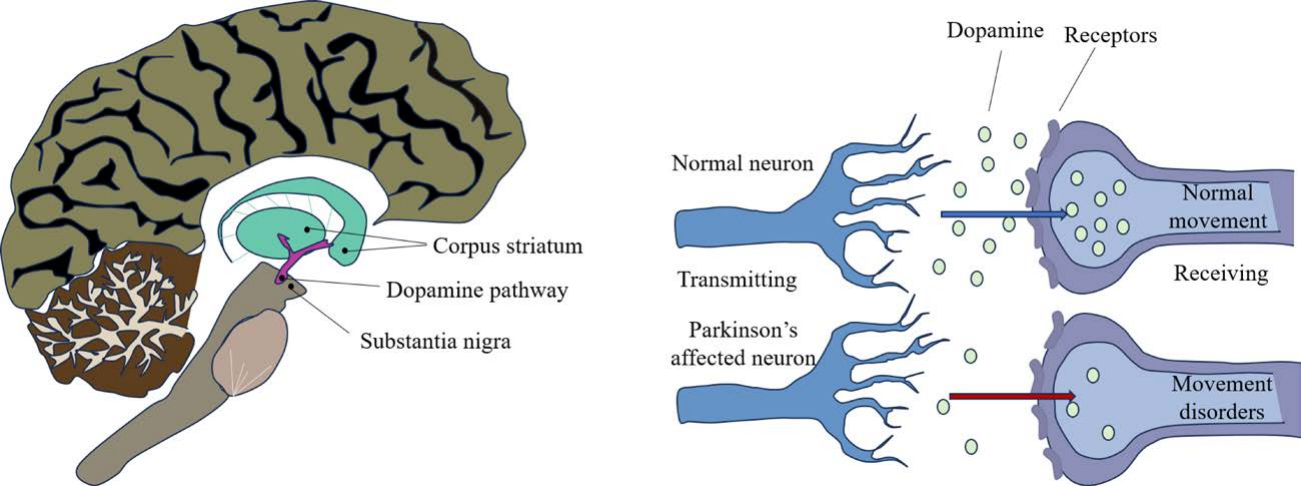
Parkinson disease is characterized by selective loss of dopaminergic neurons originating in the substantia nigra. The accumulation of alpha-synuclein in neurons results in the presence of Lewy bodies and Lewy neurites as well as neuroinflammation in the brain. The degeneration of dopaminergic neurons and decreased dopamine levels can cause primary motor symptoms.

Parkinson disease (PD) is hallmarked by the loss of dopaminergic (DA) neurons in the substantia nigra (SN). Aberrant accumulation of alpha-synuclein (α-syn) in neurons leads to the formation of Lewy bodies and Lewy neurites, and induces neuroinflammation in the brain.^[1–3]^ The degeneration of DA neurons and the consequent reduction in dopamine levels can lead to primary motor symptoms, including resting tremors, bradykinesia, stiffness, and gait disorders. Non-motor symptoms linked to this disease encompass autonomic nervous system dysfunction, cognitive impairments and psychiatric manifestations, such as anxiety, depression, and apathy – along with sleep disturbances like insomnia.^[1,4–6]^ The pathogenesis of Parkinson is illustrated in Figure 1.

Neuroinflammation acts as a native immune response that protects neurons from injury and facilitates repair of damaged neurons. However, its neurotoxic effects exacerbate neuronal damage. Neuroinflammation arises from the complex interactions between various cell types in the brain. Neurons, astrocytes, microglia, and endothelial cells are susceptible to α-syn aggregation through mechanisms such as phagocytosis, endocytosis, and Toll-like receptor stimulation. Consequently, these interactions disrupt cellular homeostasis, trigger the release of proinflammatory mediators, and upregulate specific receptor expression.^[7]^ α-syn aggregates are capable of acting as autoantigens that induce T cell-mediated neuroinflammation and ultimately lead to the degeneration DA neuronal.^[8]^ Additionally, neuroinflammatory responses are regulated by immune cells, including glial and peripheral immune cells, as well as cytokines and chemokines.^[9–11]^ The overexpression of interferon-γ (IFN-γ), interleukin (IL)-6, and IL-1β in the brain can trigger neuroinflammatory responses. However, elevated levels of peripheral cytokines disrupt the structure and function of the blood–brain barrier (BBB). Once the BBB breaks down, inflammatory cells, immunoglobulins, and complement components present in the blood gain to infiltrate the brain. This exacerbates neuroinflammation and neural injuries.^[12]^

Proinflammatory cytokine levels are significantly increased in the circulation of PD patients. The increase in these inflammatory cytokines is hypothesized to stem from T cell activation.^[13]^ There is growing evidence that peripheral immune cells are fundamental to disease development and that T cells are involved in α-syn-related neuroinflammation in PD.^[2,12]^ In recent years, changes in CD4+ and CD8+ T cells and their subsets have been identified in the SN of patients with PD and various preclinical models of PD.

## 2. Immunomodulatory role of CD4 + T lymphocytes

### 2.1. The connection between autoimmunity and PD pathology

In the context of physiological dysfunction, the structural integrity of the BBB is compromised by the combined effects of oxidative stress, chronic inflammation, aberrant protein deposition, and vascular dysfunction.^[14]^ Impaired BBB integrity in PD allows circulating T lymphocytes from the periphery to invade brain tissue. Recognition of α-syn presented by major histocompatibility complex class I (MHC-I) on neurons and MHC-I on microglia leads to T cell activation and release of proinflammatory cytokines. Under persistent inflammation and cytotoxic conditions, DA neurons ultimately undergo cell death.^[15–17]^

Activated microglia and astrocytes during neuroinflammation drive the production of proinflammatory cytokines, and infiltration of peripheral immune cells into the central nervous system (CNS). Various cytokines released by microglia and astrocytes can induce neuroinflammation, thereby leading to neurodegenerative changes.^[18–20]^ NF-κB is one of the most important regulators of the inflammatory immune response. When NF-κB is activated, it induces the synthesis of IL-1 family cytokines and amplifies inflammatory signals through the progressive release of proinflammatory mediators in glial cells.^[19,21]^

Inflammation promotes the recruitment of immune cells and activates the JAK/STAT pathway, which plays a key role in triggering innate immune responses, mediating adaptive immune responses, and modulating inflammatory reactions. Dysregulation of the JAK/STAT pathway has been associated with various autoimmune and neuroinflammatory diseases. Targeting the JAK/STAT signaling pathway may exert neuroprotective effects against α-syn-induced neuroinflammation and DA neuron degeneration.^[22]^

### 2.2. The relationship between the balance of peripheral blood CD4 + T lymphocyte subsets and PD

With CD4 + effector subsets, T helper (Th) cell 1 and Th17 cells facilitate a proinflammatory response, whereas Th2 and regulatory T cells (Treg) cells elicit anti-inflammatory and immunosuppressive responses. The equilibrium between proinflammatory and anti-inflammatory immune responses is indispensable for the preservation of systemic homeostasis, particularly in the CNS, where dysregulation may precipitate neurodegenerative diseases.^[11,23]^ Experimental evidence has demonstrated that in 6-hydroxydopamine-PD rats, a large number of CD3+, CD4+, and CD8 + T lymphocytes migrate from the blood vessels to the SN with the intent of targeting DA neurons.^[24]^ Consequently, these lymphocytes may therefore serve as promising biomarkers for assessing disease progression in PD. The current investigation revealed the presence of CD8 + and CD4 + T cells (but not B cells) in both postmortem human tissue specimens and mouse models of PD induced by 1-methyl-4-phenyl-1,2,3, 6-tetrahydropyridine (MPTP), infiltrating the brain parenchyma in the context of PD.^[20,25]^ Findings from animal model studies indicate that CD4 + T cells are pivotal in determining the extent of DA cell death mediated by T cells.^[26–28]^

## 3. T cells and cytokines

### 3.1. CD4 + T lymphocyte subsets

**Figure 2.**
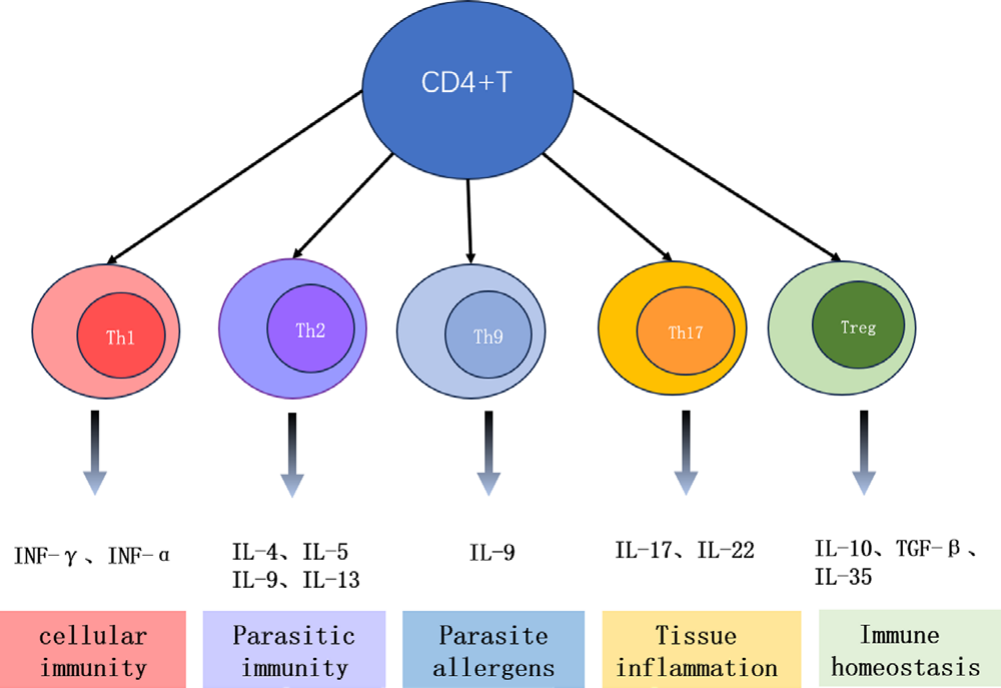
CD4 + T lymphocyte subsets and there cytokine production and main functions. T helper (Th) Th1 cells produce interleukin (IL)-2, interferon-γ (IFN-γ), and tumor necrosis factor-α (TNF-α) and are mainly involved in delayed hypersensitivity and cell-mediated immune responses, such as autoimmune diseases. Th2 cells are known to producing the cytokine interleukin (IL)-4, which mediates host defense against worms and has been implicated in the pathogenesis of allergic diseases. Th17 cells mainly secrete IL-17, which plays a crucial role in the proinflammatory properties, inflammation, and protection of the host from extracellular bacterial and fungal infections. Treg cells secrete IL-10 and transforming growth factor (TGF-β).

According to their effector functions and MHC recognition patterns, T lymphocytes are further subdivided into helper T cells (CD4+) and cytotoxic T cells (CD8+).^[4,13,29,30]^ CD4 + T cells may accelerate the pathological progression of PD by enhancing the secretion of proinflammatory factors and inducing DA neuronal toxicity through activation of the Fas–Fas ligand (FasL) apoptotic signaling pathway.^[15]^ CD4 + T cells were initially divided into 2 groups based on their effector functions: CD4 + T cells that may acquire proinflammatory phenotypes, such as Th1 and Th17, and anti-inflammatory phenotypes, such as Th2 and Tregs.^[25,26,31]^ However, scientists have realized that other subsets of CD4 + cells, such as Th9 and Th22 cells.^[32]^ Th9 cells secrete IL-9; Th22 mainly secretes cytokines, such as IL-22, IL-26, and IL-13.^[11,13]^ These cytokines mediate neuronal death, activate neurotoxic glial cells, promote macrophage and B cell infiltration into the brain, and strengthen the cytotoxicity of proinflammatory T cells toward DA neurons. Cytokines and their functions are shown in Figure 2.

### 3.2. General changes in T cells

Impairment of lymphatic vessels or the BBB has been observed in patients with PD, suggesting that the distribution of T cells in the brain mirrors the distribution of T cells in the peripheral blood. Analysis of circulating lymphocytes in the blood of patients with PD revealed significant changes in the frequency of lymphocyte subsets.^[11]^ We found that the initial CD4^+^ and CD8^+^ T cell counts in patients with PD were significantly reduced.^[26]^ Most studies confirmed that CD4^+^ T cells were reduced in PD patients and declined further with disease progression.^[11,29,32–34]^ This reduction was mainly attributed to the decrease in Th17, Th2, and Treg cells, but not the Th1 subset.^[13,31]^ Calopa et al reported that CD4^+^ T cells in PD patients showed increased susceptibility to apoptosis, which also contributed to their reduced count.^[13,35,36]^ As reported by Chen et al, PD patients exhibited higher percentages of CD3^+^ and CD4^+^ T cells in their peripheral blood compared with healthy controls, whereas the percentage of CD8^+^ T cells remained relatively stable; this resulted in a significant increase in the CD4/CD8 ratio among PD patients. Therefore, the peripheral blood T cell proportion in PD patients might be linked to differences in PD sample sizes and ethnic backgrounds.^[37]^

#### 3.2.1. Changes in T cells are based on the early and late stages of PD

Hu et al reported that the percentages of CD4^+^ and CD8^+^ T lymphocyte subsets, together with the CD4+/CD8+ ratio, were significantly elevated in patients with advanced PD compared with those at early stages of the disease.^[38]^ The proportion of circulating CD8+ T cells was markedly lower in late-stage PD patients (Hoehn–Yahr stages 4–5) than in control subjects, suggesting that immune dysfunction is tightly linked to the progression of PD.^[39]^

#### 3.2.2. Changes in T cells are based on gender or genetic differences

This study demonstrated no significant differences in the proportions of CD3+, CD4+, and CD8 + T lymphocyte subsets, as well as the CD4+/CD8 + ratio, between female and male patients.^[39]^

Pink1 and Parkin cooperatively regulate mitochondrial quality control, and their loss-of-function mutations contribute to familial PD. Pink1/Parkin deficiency leads to abnormal peripheral lymphocyte subsets, characterized by increased CD4^+^ T cells and decreased CD8^+^ T cells and B cells.^[40]^

### 3.3. Cytokines

Changes in the T cell population can also lead to changes in circulating cytokines. In the past 15 years, several other studies have described special peripheral immune changes in PD, including a reduced CD4^+^/CD8^+^ T cell ratio and elevated IFN-γ- and IL-4-producing T cells.^[9,28]^ Th1 and Th17 + CD4 + cells promote an increase in IFN-γ, tumor necrosis factor-α (TNF-α), and IL-17 levels.^[17]^ Their increased numbers are associated with higher IL-6, IL-17, and lower IL-10, transforming growth factor-β (TGF-β) in serum,^[12]^ while Treg cells show no significant change.^[41]^ In PD patients, the levels of proinflammatory cytokines in the blood are increased, such as IL-2, IL-6, IL-8, monocyte chemotactic protein-1, TNF-α, and IFN-γ. Such increases in proinflammatory cytokines and chemokines indicate the activation of immune responses, supporting the notion that peripheral inflammation contributes critically to the progression of PD.^[17,42]^

In summary, the abnormal changes in serum inflammatory factors and peripheral T cells underlie immune dysregulation in PD, implying that these parameters could act as predictive biomarkers in clinical settings.^[24]^

## 4. The role and changes of CD + T lymphocyte subsets

### 4.1. Th17 cells

Th17 represent one of the most extensively investigated CD4^+^ T helper subsets.^[33]^ Their differentiation relies on the induction of several cytokines, such as IL-6, IL-23, IL-1β, TGF-β, and IL-21.^[43]^ RORγt is an indispensable transcription factor that mediates the biological function of Th17 cells.^[44]^

#### 4.1.1. Pathogenicity of Th17

On the one hand, Th17 cells directly induce neuronal apoptosis through Fas/FasL interactions. Through the secretion of IL-17A, Th17 cells are capable of eliciting cytotoxic effects on neurons.^[41]^ Numerous studies have shown that IL-17A plays a pathogenic role in the CNS by stimulating glial cells and enhancing neuroinflammatory responses.^[45]^ However, these cytokines lead to the overactivation of glial cells, which in turn release more inflammatory mediators, such as TNF-α, IL-1β, granulocyte-macrophage colony-stimulating factor(GM-CSF), and inducible nitric oxide synthase,^[46]^ as well as fewer neurotrophic factors. Th17 cells release inflammatory cytokines that promote glial cell activation.^[47]^ Overactivation of glial cells leads to disorders in inflammatory mediators and neurotrophic factors, leading to neuroinflammation and neurodegeneration.^[48,49]^

#### 4.1.2. Th17 cells damage DA neurons through intercellular contact mechanisms

Shi et al described the exact mechanism by which Th17 cells in PD mediate DA neuronal death through the secretion of IL-17A.^[43,45]^ Th17 cells exacerbate MPP+-induced functional neuronal loss by promoting glial activation in vitro. Researchers have relied on the intercellular (Th17-neuron) contact mechanism of interactions between adhesion molecules expressed on the cell membrane to demonstrate the direct damage of Th17 cells to DAergic neurons. Reynolds and colleagues have shown that Th17 cells exacerbate nigrostriatal pathology.^[50]^

In addition to promoting the secretion of various proinflammatory cytokines (IL-17A, IL-6, IL-23, and IL-1β) to participate in PD pathogenesis, Th17 cells can also directly induce DA neuronal loss via interactions with specific membrane proteins, including leukocyte function-associated antigen-1 and intercellular adhesion molecule-1.^[51]^ Th17 cells can directly induce apoptosis in neurons via the Fas/FasL interaction. IL-17, IL-21, IL-22, IL-23, IFN-γ, and GM-CSF are Th17-derived cytokines that directly or indirectly enhance inflammatory reactions in the CNS through other immune cells.^[48]^ IL-17-producing cells induce cell death in midbrain neurons. Binding of IL-17 to its receptor (IL-17R) on neurons leads to altered activation of NF-κB and subsequent neurodegeneration.^[43]^

Th17 cells infiltrate the brain and activate microglial cells. IFN-γ produced and secreted by activated microglia promotes abnormal upregulation of MHC class I expression in catecholaminergic neurons.^[43]^ All these processes play crucial roles in neuroinflammation.

#### 4.1.3. Changes of Th17 cells

An in vitro study showed that increased numbers of Th17 cells in the blood of patients with PD are associated with the death of human induced pluripotent stem cell-derived midbrain neurons. The level of Th17 cells in PD patients is significantly higher than that in healthy controls.^[4,17,52]^ Another study also found a significant correlation between Th17 cell levels and parts I and II of the MDS Unified Parkinson Disease Rating Scale. Kustrimovic et al found that Th17 cells in the blood of patients with PD decreased, whereas other studies found that Th17 cells in the peripheral blood of patients with PD increased.^[13,25]^ Elevated levels of circulating Th17 cells have been observed in the early stages of PD.^[51,53]^ The different changes in Th17 cells are caused by various factors.^[54]^ Therefore, further research is required to determine the exact changes in Th17 cells.

In summary, Th17 cells damage the CNS and induce neuroinflammation. Targeting Th17 cells represents a promising therapeutic strategy for slowing neurodegenerative progression in PD.^[55]^

### 4.2. Th1 cells

#### 4.2.1. The role of Th1 cells in inflammatory response

Th1 cells mainly mediate immune responses to extracellular pathogens. Differentiation of CD4 + T cells into Th1 cells is primarily driven by the key cytokines IL-12 and IFN-γ.^[42]^

First, IFN-γ and TNF-α secreted by Th1 cells can amplify NF-κB signaling activation in microglia. In addition, IFN-γ promotes the secretion of chemokines from neurotoxic microglia. Among them, CXCL16 binds to its receptor CXCR6, thereby facilitating the infiltration of Th1 and T cell toxic 1 cells. This interaction further activates the NF-κB pathway in Th1 cells, which in turn elevates the transcription of proinflammatory genes and reinforces T cell-driven inflammatory reactions.^[9,15]^

When APCs are activated, IL-12 is released. APCs-derived IL-12 and NK cell-produced IFN-γ activate transcription factors and signal transduces activators (STAT4) and 1. This signaling cascade promotes the expression of T-bet (T-box expressed in T cells) in T lymphocytes, thereby driving the polarization of CD4 + T cells toward a Th1 phenotype.^[42]^ Th1 cells exert dual pro-inflammatory effects in PD pathogenesis. On the one hand, they stimulate microglia to release inflammatory mediators that promote DA neuron damage. On the other hand, they promote the transcription of the c-mer proto-oncogene tyrosine kinase gene in macrophages, thereby enhancing the phagocytic activity of microglia. c-mer proto-oncogene tyrosine kinase is a phagocytic receptor that aggravates damage to DA neurons by releasing effector molecules into the microglia. During inflammation, microglial phagocytosis of DA neurons promotes the pathological progression of PD, potentially contributing to the loss of DA neurons.^[15]^

#### 4.2.2. Th1 changes

In patients with PD, Th1 cells that produce IFN-γ are significantly elevated compared with IL-4 producing Th2 cells.^[11,28,35]^ The enhanced Th1 response indicates that cellular immunity is in an active state and host cells defend themselves against infection by intracellular pathogens.^[56]^ However, another study showed an increase in Th1 cells and cells in patients with PD and a corresponding decrease in Th2 and Th17 cells.^[57]^ This imbalance of pro-inflammatory cells (mainly Th1) may be a persistent factor in neuronal degeneration caused by neuroinflammation.

### 4.3. Th2 cells

Th2 cells also exhibit anti-inflammatory and protective properties. Functioning as key mediators of humoral immune responses, Th2 cells protect against helminth infections, facilitate tissue repair, and contribute to chronic inflammatory conditions including asthma and allergies. The main regulators of Th2 differentiation are IL-4 and IL-2.^[42,58]^

#### 4.3.1. Role of Th2 cells

Th2 cells differentiate into naive T cells under the influence of IL-4 and GATA-3 transcription factors.^[44]^ In addition, IL-5 activates eosinophils, which release various proinflammatory and cytotoxic proteins. IL-4 and IL-13 released by Th2 cells induce the expression of IL-1 receptor antagonist, block the entry of IL-1β into microglia, and further reduce the number of neurotoxic microglia and release of inflammatory factors and chemokines.^[15]^

#### 4.3.2. Changes of Th2 cells

Luquin et al showed that Th2 cell counts in patients with PD did not differ significantly from those in controls; however, a significant increase in IL-13 was observed.^[59]^ In contrast, other studies have reported lower absolute numbers and frequencies of Th2 cells in patients than those in healthy subjects. Sulzer et al observed that alpha-syn peptide mainly activates Th1, which produces IFN-γ, and Th2, which produces il-5. Consistent with the decrease in DA activity in patients with PD, the proportion of Th2 cells was decreased.^[60]^ The prevalence of pro-inflammatory phenotypes in PD is related to the anti-inflammatory response induced by Th2 cells. Therefore, the effect of Th2 on PD is a therapeutic target and immune strategy for PD.^[61]^

### 4.4. Th9 cells

#### 4.4.1. Function of Th9 cells

Th9 cells are a new subgroup of T cells discovered in recent years that mainly express the cytokine IL-9,which has immune functions. The balance of cytokine signaling is essential for the induction of Th9 cell development and differentiation.^[62]^ The development of Th9 cells requires co-induction of TGF-β and IL-4.^[63]^ Th9 cell differentiation and IL-9 production are modulated by a complex cytokine network: IL-1, IL-2, IL-10, IL-21, IL-25, and TNF-α.^[63]^

#### 4.4.2. Transcriptional regulation of Th9 cell differentiation

IL-4, TGF-β1, IL-2, and T cell receptor stimulate the expression of downstream transcription factors, including BATF, SMAD3, TAK1-SIRT1-mTOR-HIF1α, PU.1, STAT5, Bcl6, NF-kB, and NFAT.^[62]^ These transcription factors interact with IL-9 promoters. Increased expression and secretion of IL-9. Downstream transcription factors of IL-4 and TGF-β, such as STAT6, GATA-3, and interferon regulatory factor 4, are necessary for Th9 cell differentiation.^[42]^ STAT5 is a downstream target of IL-2, which directly binds to the IL-9 site, thus promoting Th9 cell differentiation. Mechanistic studies have shown that IL-2-STAT5 signaling can promote Th17 cell generation and Th9 cell differentiation. Thus, each transcription factor may play an important role in the expression and production of IL-9 in CD4 + T cells.^[62]^

Taken together, Th9 cells appear to be pathogenic. Therefore, blocking the IL-9 pathway may be a promising strategy to alleviate the immunopathology of autoimmune and inflammatory diseases.^[46]^

### 4.5. Treg cells

#### 4.5.1. Origin of Treg cells

Regulatory T cells, the major immunoregulatory cell population, express DA receptors and exert neuroprotective effects in mouse models of PD. First, it is directly produced during the development of thymus T cells in early life.^[64]^ Second, under the influence of TGF-β, it differentiates from naive CD4 + T cells in peripheral blood, which is necessary for its function.^[65,66]^ Tregs have immunosuppressive properties that can inhibit the activation, proliferation, and effector functions of T cells, natural killer cells, and antigen-presenting cells both in vitro and in vivo.^[67]^

#### 4.5.2. Role of Treg cells

Tregs are a subgroup of immunosuppressive T cells and key regulators of immune cell tolerance.^[66,68]^ In CNS, its immunoregulatory role involves suppressing IFN-γ and TNF-α secretion by inactivating immune cells and releasing IL-10 to control acute inflammation.^[58,69]^ Under normal physiological conditions, Tregs inhibit the inflammatory response of effector T cells. In the pathological state, the anti-inflammatory effects of Treg cells are diminished, leading to the persistence of the proinflammatory environment. Tregs primarily maintain immune homeostasis and tolerance in neurodegenerative diseases by inhibiting immune activation.^[32]^ Treg cells maintain this balance by exerting an inhibitory effect and blocking proinflammatory responses.^[13]^ Tregs not only inhibit the effector immune response but also transform Th1 and Th17 responses into neuroprotective responses, suggesting that Tregs have a potential role in PD.^[11,61,70]^

Several animal studies have confirmed that Tregs play an important regulatory role in the progression of PD. First, in an MPTP PD model, Treg deficiency significantly enhances neuroinflammation and accelerates neurodegenerative changes.^[11]^ In a mouse model of cerebral infarction, Tregs accumulate in the brain during the chronic phase and achieve neural repair through interaction with astrocytes.^[71]^ During the acute phase of experimental stroke, Treg expansion exacerbates brain injury.^[72]^ Second, in a PD model, nitration of α-synuclein (N-α-syn) elicits an adaptive immune response to new antigenic epitopes, thereby intensifying neuroinflammation and nigrostriatal degeneration. We found that this neurodegenerative activity is largely due to Th17 cell-mediated CD4 + CD25 + Treg dysfunction. Tregs can not only prevent MPTP-induced DA degeneration but also regulate the inflammatory response after MPTP poisoning. Preclinical studies have shown that CD4 + regulatory T cells can control microglial inflammatory activity and reduce the loss of DA neurons.^[73]^ However, the use of the nitrated form of alpha-SYN as an immunogen induces a deep-effect T cell (Teff) response, which exacerbates neuroinflammation and neurodegeneration. Taken together, these results suggested that nitrated form of α-syn immunologically induces Th1 and Th17 T cells and causes Treg dysfunction. Th1, Th17, and Tregs play an irreplaceable role in MPTP-induced inflammatory response and DA neuronal death.

Tregs modulate immune responses by inhibiting microglia activation and inducing microglia phenotypic transition. In general, Tregs may suppress innate and adaptive proinflammatory and neurotoxic immune responses. Tregs slow neurodegeneration and repair damaged neurons by regulating homeostasis and neurotrophic immune response. These strategies may help slow or stop the progression of PD.^[11]^ In summary, Tregs protect neurons from injury induced by overactivated neurotoxic microglia by promoting the apoptosis of these cells and reducing their numbers.^[15]^ The animal model also verified the hypothesized mechanism of Treg control of neuronal destructive immunity induced by N-α-syn, which provides a good theoretical basis for the formulation of PD immune strategies in the future.^[74]^

#### 4.5.3. Changes of Treg cells

Some reports have shown that Tregs, which maintain active immune tolerance and reduce inflammatory responses are significantly reduced in patients with PD, whereas activated pro-inflammatory myeloid and effector T cells are increased.^[66]^ Bas et al reported that the Treg frequency was reduced in patients.^[75]^ Numerous studies have analyzed the differences in T cell subsets between patients and healthy individuals. Patients had fewer CD4 + Th cells and more Tregs than the age-matched healthy controls. Variations in Treg levels in individuals with PD may be explained by the use of different phenotypic approaches and diverse patient recruitment protocols.^[17]^ Williams et al observed significantly increased expression of IFN-γ and IL-10 in Treg cells 4 weeks after injecting adeno-associated virus 2-synuclein (syn) into mice. These results suggest that immune activation is significantly enhanced in the early stages of PD,^[20]^ indicating that Treg cells may be involved in the regulation of inflammatory responses during the early phase of the disease. Nevertheless, Treg cell function in controlling brain inflammation gradually diminishes as PD advances. This alters the balance between pro- and anti-inflammatory cells and disturbs overall immune homeostasis.^[15]^

While Treg cells are critical in maintaining autoimmune tolerance and suppressing harmful immune responses, they may also inhibit protective T cell responses. Importantly, growing evidence suggests that Treg activity may be suppressed or dysfunctional in certain neurological disorders, which may aid in the study of this disease. As Tregs offer highly specific and potent immunosuppressive capabilities, they may be used as therapeutic agents for neurological pathologies in the future.^[67]^

## 5. Th1/Th17 and Th1/Treg imbalances are involved in the PD process

Th1 and Th17 cells are major cellular mediators responsible for immune-mediated damage. Proinflammatory factors secreted by activated Th1 and Th17 cells can modify the biological behavior and immune function of other infiltrating cells in the inflammatory microenvironment, ultimately affecting the survival and differentiation of these cells.^[48]^

Studies have reported that Th17 levels and Th1/ Th17 in the peripheral blood of PD patients are significantly higher than those of Th cell subtypes, further supporting the prominent role of proinflammatory Th1 and Th17cells.^[55]^ Chen et al suggested that the peripheral immune system plays an active role in the progression of PD, and concluded through the blood analysis of PD patients that the proportion of Th1 and Th17 cells increased, while Th2 and Treg decreased.^[11,76]^ Similarly, the number of Th1 cells in the blood of experimental PD mice increased and the number of Treg cells decreased. It is suggested by these data that the imbalance between Th1 and Treg cells plays a critical role in PD pathogenesis.^[70]^ In addition, the Treg/Th17 ratio in female PD patients differs from that in female controls, highlighting the importance of investigating the Treg/Th17 ratio.^[32,77]^

Although several studies have investigated Th1 and Th17 cells, their findings are inconsistent, and further in-depth research is required to draw reliable conclusions.

## 6. Immunotherapy targeting CD4 + T cell-related cytokines

Local inflammation and adaptive immune responses are associated with neuronal death. Owing to the significant role of inflammation in PD, small-molecule drugs that specifically target inflammatory process, particularly antagonists of proinflammatory factors, have been identified as potential treatments for PD.

### 6.1. Interleukin-1 receptor

Anakinra (Kineret) is a recombinant antagonist of the human IL-1 receptor, which exerts its inhibitory effect on IL-1 by targeting IL-1 type 1 receptors. Studies have demonstrated that tissue cytokine profiling reveals markedly elevated levels of IL-1β. In a rat model of PD induced by 6-hydroxydopamine, anakinra significantly decreased TNF-α and IFN-γ levels and attenuated DA neuronal loss.^[42,78]^

### 6.2. IL-17A receptor

In this context, secukinumab acts as an inhibitor and has neuroprotective effects.^[42]^ Recent studies have demonstrated that IL-17A accelerates neurodegeneration in PD by activating microglia and recruiting peripheral immune cells including Th17 cells into the brain. Secukinumab can alleviate neuroinflammation in the CNS via decreasing immune cell invasion and downregulating chemokine expression. These results suggest that secukinumab represents a promising therapeutic strategy in PD, highlighting the necessity for further investigations to optimize clinical use and expand its application in other neurodegenerative diseases.^[42,79–81]^

### 6.3. RORγt

RORγt is an essential transcriptional regulator that drives Th17 cells differentiation and IL-17 secretion, which is crucial for neuroinflammation related to PD. Inhibition of RORγt restricts the polarization of Th17 lymphocytes and lowers the release of various proinflammatory mediators, thereby weakening the overall inflammatory reaction. This process may effectively attenuate PD-related neuroinflammation and protect DA neurons from damage, providing a potential immune-based strategy for delaying disease progression.^[42]^

### 6.4. GM-CSF

Across both animal and clinical research settings, GM-CSF functions as the key inducer of Tregs. As an inflammatory cytokine, it exerts its effects primarily by regulating the differentiation of tolerogenic dendritic cells, thereby increasing Treg numbers and augmenting their functional capacity. Currently, GM-CSF is undergoing clinical trial assessment; more specifically, a randomized, double-blind, phase 1 trial involving patients with PD showed that daily treatment with sargramostim – a recombinant form of human GM-CSF – for a 2-month period effectively improved patients’ cortical motor function while also increasing Treg counts.^[42]^

## 7. Conclusions

CD4^+^ T cells are critically involved in the degeneration of DA neurons. As major immune cells, they comprise several subsets. These subsets interact via cytokine secretion to form a regulatory network that preserves immune homeostasis and physiological function. The balance of the T cell subpopulation is vital for the regulation of neuroinflammation. The disruption of this balance can result in tissue damage and other pathological conditions. Research has shown that CD4^+^ T-cell subsets is abnormal in PD patients, indicating an altered immune response. The abnormal proportion of peripheral T-cell subsets may help evaluate disease progression in PD patients, making it a potential marker for the clinical diagnosis and prognosis of this disease.

Further research is necessary to understand the role of T cell subsets and their dysfunctions in degenerative diseases. Given the significant impact of neuroinflammation on the progression of PD, immunotherapy targeting CD4 + T cells has become a promising approach for alleviating the disease. A deeper understanding of peripheral adaptive immune regulation in PD will lead to the discovery of effective immune regulatory treatments.

## Acknowledgments

We thank LetPub (www.letpub.com) for its assistance.

## Author contributions

**Conceptualization:** Le Wang, Ruijun Su.

**Funding acquisition:** Ruijun Su.

**Investigation:** Le Wang.

**Methodology:** Le Wang, Ruijun Su.

**Writing – original draft:** Le Wang.

**Writing – review & editing:** Ruijun Su.
